# Supplementary material for: Rock Steady Boxing: A qualitative evaluation of a community exercise program for people with Parkinson’s disease
Source: PLoS One. 2024 Dec 19;19(12):e0309522. doi: 10.1371/journal.pone.0309522 (PMC11658574; doi:10.1371/journal.pone.0309522)
Supplement: S1 File — (DOCX) [file pone.0309522.s001.docx]

**Supporting information**

S1 Fig: Demographic intake form


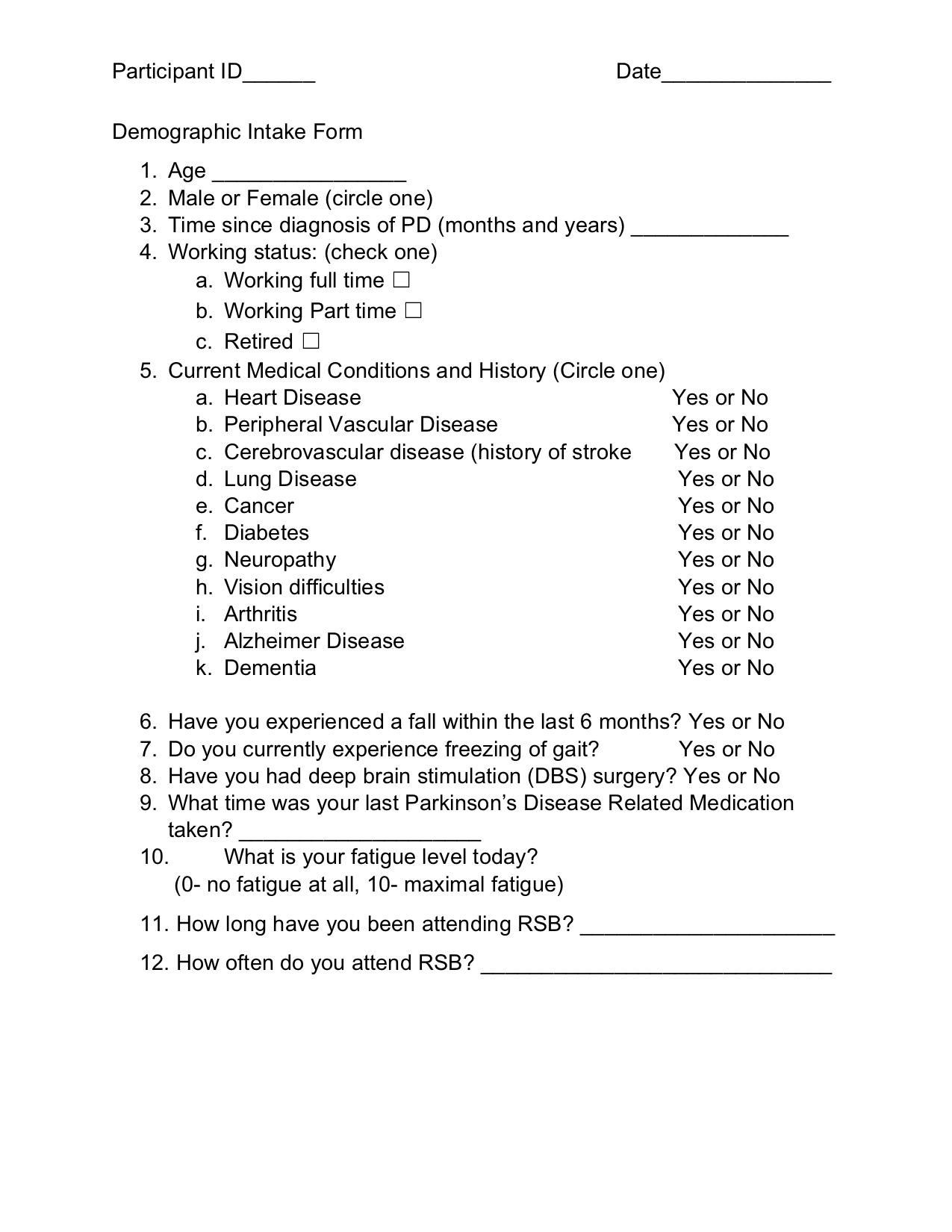


S2 Fig: Structured observation form

Date/Time:

Researcher Observer Name:

1. Description of class location:
2. Description/Number of participants:
3. Structure of Class/class components:
4. Types of Activities (stretching, movement activity, breathing, other):
5. Interaction (teacher-participant, participant-participant, etc.) :
6. Barriers and Facilitators to Activities and Interactions:
7. Safety Factors (+/-):
8. Other Notes:

S1 Table: Semi-structured interview guide

| **Interview Question** | **Study Goal** |
| --- | --- |
| How did you find out about this program? | 1B, 1C, 1E |
| Why did you decide to come to the program for the first time? | 1B, 1C, 1E |
| What were your goals in starting this program? | 1B, 2A |
| Why did you decide to continue to participate in the program? | 1B, 1C, 2A, 2B, 2D, 2E |
| How do you usually get here? (transportation - is this a reason they cancel if transportation is unavailable?)   - What barriers or challenges do you face in participating in this program? How do you overcome these? | 1A, 1C, 1E |
| On days when you do not come to the class, what might the reason be?   - What makes it hard to participate? | 1A, 1D, 1E |
| Describe the experience of exercising with other PwPD (compared to people without PD). Do you spend time with other fighters outside of class? | 2D, 2E |
| What were your feelings about exercise prior to starting this program? How have they changed since you have been participating? | 1C, 3A, 3C |
| If they indicate they have a care partner on PDQ-39: How does your care partner support you? Are there ways they could be more supportive? | 1C, 1D |
| Has this program changed the way you interact with your friends/family/colleagues? How so? | 3A, 3B |
| In what ways does the instructor influence your participation in the program?   - Do you choose which class you attend based on the instructor? What are the qualities of the instructor that you find helpful? | 2A, 2D |
| How do you feel during class? (Physically, mentally, safety)   - Has this changed over time? | 2B, 2C |
| How do you modify an activity that is too easy or too hard during class? | 2A, 2C |
| How do you interact with other participants during class? With the instructor? | 2D |
| Have you participated in other group exercise programs?   - If yes: how is this program different from those? | 2E |
| How do you feel after class? Mentally, physically, otherwise   - Right after class: - Later in the day: - Do you notice any changes in mental status? Fatigue? Apathy? | 3A, 3B, 3C |
| How has the program impacted your level of independence? At home, at work, in the community, etc? | 3A, 3B, 3C |
| What sorts of other hobbies or jobs did you have prior to your diagnosis of PD? | 1B, 1C |
| In what ways (if any) has the program impacted your movement or mobility outside of class?   - Do you notice any differences in your gait, your ability to go from sit to stand, your tremors, etc. - Have any activities gotten easier or harder? | 3A, 3B, 3C |
| If you could make changes to the program, how would you change it? | 2A, 2B, 2C |
| Do you have any additional questions, comments, or concerns about the program?   - Are there benefits from this program or things that you take away from the class that we haven’t discussed?’ | N/A |

| S2 Table: Additional participant quotations for all result themes | | |
| --- | --- | --- |
| **Global Themes** | **Organizing Themes** | **Quotes** |
| **Contextual Factors: environmental** | Hearing about RSB from a Trusted Source |  |
|  | RSB as a Priority in Participant’s Schedules | ***Participant 2 Care Partner: “****We try to schedule around so we make sure to come here”*  ***Participant 5: “****No, ‘cause I schedule it and I block it”*  ***Participant 8:*** *“Well, occasionally something comes up, but we’ve pretty much blocked out those time frames.”* |
|  | Family/Care Partner Support | ***Participant 4:*** *“she [my wife] gets on me about not—when I’m not exercising sometimes and I need to…she supports…encouraged me to start it to start with...she gets me here…and I can tell that she wants me to improve…or maintain…mainly what I’m concerned about is maintaining and…not regressing...She is very supportive, gets after me when I’m not—when I’m laying around too much.”*  ***Participant 6:*** *“I was bitter...I was angry...and then, with the help of my wife and my family and all that, and my friends, I snapped out of it...I’m where I am today, and then I joined Rock Steady. And the combination of all that has gotten me to where I’m able, I’m at a point where I can function in life and still realize that I got a lot of years ahead of me, and uh, and you don’t die with, from Parkinson’s you die with it. It’s not going to kill me, and I’m here to inspire others that have it.”* |
|  | Transportation Available | ***Participant 6: “*** *I—I—for short distances I drive myself, like I drove myself today. Um, I don’t drive when I don’t have to because my foot gets, when I’m stopped at a stop light, and I’ve um, got a foot on the break, sometimes it feels like it’s getting weaker, like I’m going to let off the break. I haven’t done it but it just feels that way. And, um, the other thing is uh, I never know, I don’t know when the tremor or the twitch in my legs might happen [In: right] and then that scares me, will I run into the back of somebody. So it’s not happening, it’s a fear in my mind that it might one day so I don’t drive if I don’t have to. But I do when necessary. “*  **Participant 10:** *“I drive. My wife comes along most of the time. But I drive...Not that I don’t want her support, it’s just that I want to try to do it as long as I can.”* |
| **Contextual Factors: personal** | Positive Exercise Beliefs and History of Exercise as Motivators | ***Participant 5****: “I used to exercise all the time. I mean, I uh, I mean I fell off some, but I would walk or jog every day. I would use isometric exercises. I’ve always exercised because I’ve always been an athlete.”*  ***Participant 8:*** “*I knew it was beneficial but in terms of overall, it’s good for everybody. But I didn’t realize or understand the specific benefits that it is for P patients by virtue of as my if I understand it correctly, 20-30 minutes of strenuous exercise helps with reduction of dopamine, which is good.”*  ***Participant 9:*** *“Well I've always been very active. I’ve always wanted to do something. I’ve always wanted to stay fit. I’ve always wanted to be strong.* *But now I feel like I don’t have an option; it’s not a choice*.*”*  ***Participant 10:*** *“Well, it made sense, ya know. ‘Cause you wanna get some sweat going and exert yourself, uh. It only helps you in the long run.* |
|  | Focused Goals as Motivators | ***Participant 1:*** *“To feel better, ya know, get more exercise in.”*  ***Participant 2:*** *“Get my golf game back.”*  ***Participant 5:*** *“My goal is to never ever use a cane, that’s my goal.”*  ***Participant 9:*** *“‘Cause I felt I needed something that was focused on what I needed. ‘Cause I didn’t know what I needed. Physical, ya know, activities, exercises, therapy, whatever, I needed it to be focused on what was wrong with me.…To slow it [Parkinson’s disease] down. Stop it, slow it down, whatever I could do to keep it at bay.”* |
|  | Overcoming Barriers due to limitations from PD  (Depression) | ***Participant 6:*** *“I was bitter. I was angry. I’m embarrassed to say that I was questioning my faith. Um…that bothered me a lot. I would go for two, two to three months straight I would go from the bedroom to my lounge chair. And I was having medication issues...and I was having side effects with the first Parkinson’s medicine. I was having side effects with the, uh, getting, with the depression medicine, getting it in me. We had to change the depression medicine. Um, I was on a—a—a medicine for depression or anxiety that was addicting, and that took me a long time to get over that. So, I don’t know if it was a combination of all that and then the diagnosis and the bitterness, but I, I was, a vegetable for—for three or four months.”*  ***Participant 10:*** *“it’s depressing because I used to do a lot of pushups. And now I can barely do 3, ya know. Uh, situps. Ya know, I used to be able to do 50 to 60, and now I do 10. Uh, that’s depressing, because I know something is happening and I have no control.”* |
|  | Overcoming Barriers due to limitations from PD  (Functional Activities) | ***Participant 1:*** *[In regards to moving from sitting to standing] “That’s probably the worst thing right there. That has, that has kind of gotten a little worse.”*  ***Participant 5:*** *“Balance and stuff, I’m not very good. And the other thing, I can do it, but I’m not real strong at getting up off my knees. I have to push up, and then sometimes I get a little push there. The other day I fell doing it.”*  ***Participant 6:*** *“But I’m going harder and I get tired and sometimes they go the full distance and I have to stop and rest. And um, that bothers me a little bit. But I know that it’s, it’s helping, but I wish I could, I wish I was in better shape.”*  ***Participant 10:*** *“I hesitate sometimes starting a conversation because I can’t select the right words. If you understand what I’m saying?...And I’m sort of drawing back at times. I’m not getting involved in the conversation, ‘cause what I wanna say I can’t say.”* |
|  | Overcoming Barriers due to limitations from PD  (Recreational Activities & work) | ***Participant 5:*** *“One thing I used to love to do was going to an antique store and buy an old ship, completely tear it down, completely rebuild it, and remake it, and um, I can’t do that now because the shaking, I can’t do the rigging anymore. And I miss that. Because that was fun.”*  ***Participant 6: “****I just couldn’t do my, my job anymore. And the stress of it. It was a very stressful job, and that made my tremors, Parkinson’s worse...they allow me to retire, medically retire they called it.”*  ***Participant 10:*** *“I used to bowl. Yeah. And now, ya know, the balance with the bowling is, is a challenge.”* |
| **Participant Experience** | Varied Class Attributes and Ability to Modify Contributing to a Positive Experience | ***Participant 1: “****Like I have to modify the punching and stuff like that...Because like my left arm, my left hand just doesn’t work right. My left arm, so it’s, you know they tell you to punch hard and I can’t punch hard.”*  ***Participant 2****: “They have modifications...for different levels. So [other participant] will sit on something, where other people are standing”*  ***Participant 3****: “I modify so it’s a little harder”*  ***Participant 9:*** *“There’s some simple little things, simple little things. There’s like this one thing that has like little screws and you put the little washers on it, and it seems stupid. But you know what, as fast as you can do it, get your little fingers moving and you do all these little things. And we had to do all these little things. It just seems silly, but those are things that people do like at a picnic or people might play here.”* |
|  | Care Partner Presence as a Necessary Component of Class | ***Participant 4:*** *“The wives will…be at each station, and um…show you what you’re supposed to do at that station if you don’t remember or… they go over it before the class starts, still it’s hard to remember which… Hard to remember without having to look…look at the board every time”*  ***Participant 10*: “**Well, she’s [care partner], she’s doing a lot for me if, if I'm having a problem putting on the, the boxing gloves, she’ll come over and help out or something like that.”  ***Structured Observation:*** *“Care partners that were present were very interactive with all participants, encouraging and assisting others besides the person they arrived with. They also assisted the instructors with setup and breakdown of materials. Care partners also provided hand-held assist or guarded individuals who were lower functioning, regardless of whether it was their partner that they arrived with...approximately ⅔ of the participants had a female care partner with them.”*  ***Structured Observation:*** *“Due to the high ratio of participants to instructors, participants did not always have direct supervision during the class. Some balance-based exercises were challenging for participants, and many appeared to struggle without instructor guidance. However, the presence of care partners partially addressed this deficit.”* |
|  | Varied Physical and Mental Responses During Class | ***Participant 5:*** *“I just push through. Um, they’re some things I get frustrated with. Like balance. But I just push through. It’s all I can do.”*  ***Participant 7:*** *“I feel like I have a lot of energy [during class].”*  ***Participant 8:*** “*About halfway through, I get real tired and the second half is kind of, is a real push...But it’s due to fatigue in my legs, weakness in my legs...that I and I just have to keep pushing or do some seated activities for a few minutes.”* |
|  | Relationships During Class Facilitate Positive Experiences and Regular Attendance | ***Participant 1: “****It was just um, make me feel better and I, I didn’t fall as much and um the friendship and stuff. It’s a really good time ya know.”*  ***Participant 4:*** *“The wives, a lot of them help out... if they didn’t have the wives there, might not have enough people.”*  ***Participant 5:*** *“Everybody is accepting, everybody knows everybody, nobody’s judging anybody. And when you come here you feel welcome, whether you want to be here or not. They all, ya know, the, the um, the Parkies, as they call themselves, all speak to each other. They’re respectful and kind.”*  ***Participant 6:*** *“I’m the loudest of the—of the group. Uh…I’ll be, I’ll be exercising and uh, doing something and right in the middle of it I’ll say, [Participant]!’ and he’ll stop and turn around, and I’ll say ‘what’s the word of the day?’ and I’ll make him say it out. And I’ll, uh, do it and I’ll—I do the counting and everything, and I’ll tell ‘em, I’ll say ‘all I can hear is [Instructor]! Better get going!’ and uh, I’ll, I’ll push em, and so I interact with them all. I don’t, I, I don’t have any, any problems with, with making them do more and get louder and all. And they do.”*  ***Participant 8: “****My experience with exercising with other patients has been good, um reinforcing and the comradery of the group. We have a lot of fun with it.”*  ***Participant 8:*** *“[The instructors] are very vivacious and I’m amazed at the energy level that they maintain...and just their excitement and enthusiasm is a positive reinforcement...they’re always high-fiving and “good job” and always encouraging, uh, participation in what we’ve done.”*  ***Participant 9:*** *“[The instructors] are great, they’re supportive. ‘Oh hey, there ya go, hit hard, yeah, yeah.’ They’re just very enthusiastic, very supportive, very happy to see you walk in the door….* *[Instructor] will say, ‘Hey, oh don’t take those gloves off, do this, ‘cause that’s too easy for you.’ No, she’ll, she’ll go around and she’ll say, ‘Ok, we’ll have one person helping somebody, one person,’ she’ll make me, she’ll pull one of those bags up and she’ll go, ‘kick’. She won’t be making anybody else kick, or tell anybody else to kick, but she’ll say, ‘You need to kick, this is too easy for you.’ Or, ‘This is too hard,’ or ‘help so-and-so do this,’ so she’s always looking at everybody, making sure that they’re being challenged at their best, where they’re at, at their best place. So, she’ll be like, a couple minutes with you, a couple minutes over here, hold a bag for somebody else, you know. And then she’ll run over here and do this, and ‘c’mon we’ll do some sit-up.’ So we’ll do some sit-ups. She’s always running around, being very active, being very aware of what everybody’s doing.”*  ***Participant 10:*** *“Well they challenge you. You go a certain amount of time in an exercise, and they push you to do a little bit more. Which is good.”* |
|  | Positive Results Contribute to Regular Attendance | See Outcomes Themes for quotes |
| **Perceived Outcomes** | Affirmation or Improvements in Exercise Self-efficacy | ***Participant 5****: “It’s affirmed, and it’s made me exercise more, maybe a little more than I would have.”*  ***Participant 6****: “This is a, this is a strong class, and um, uh, I feel like I’ve accomplished something. I feel I’ve got a good workout, and I am tired and sweat like crazy...it’s a good workout so yeah, I feel good but I’m also, I feel like I’ve accomplished something and I’ve got a workout. I’m tired.”* |
|  | Functional Improvements in Gait and Balance but other Physical and Mobility Symptoms Remain | ***Participant 1:*** *“I haven’t fallen as much. I used to fall every, I used to fall five or six times a week. Now I fall maybe once every two weeks.”*  ***Participant 2 and Care Partner:***  *Care Partner:” I will say that he did start the program uh using a walker.”*  *Interviewer: “I heard that about him.”*  *Care Partner: “Yeah, and so he’s you know progressed where he’s walking in now.”*  *Interviewer: “So, do you still use the walker at all?”*  *Participant 2: “No”*  *Care Partner:” Not here.”*  ***Participant 5****: “I can do exercises easier now than I could then. When I first came here I think my hand-eye coordination was not as good as it is now.”*  ***Participant 6*:** *“Like I’ve said, I’m still a one or a two [Referencing to Hoehn & Yahr stages], so I haven’t really not been able to do anything I wanted to do...uh, has the class made a difference, would I have gotten to the point where I couldn’t do something and the class has stopped the progression of that? It’s hard—I don’t know. I can’t say.”*  ***Participant 8****: “It gives me a little more steadiness, a little more strength and endurance.”* |
|  | Fatigue or Increased Energy After Class | ***Participant 4:*** *“After my nap, at the house…spend 2 hours…rest and…get back to doin’ things.”*  ***Participant 9****: “I always feel good when I leave here. I really do. I always feel good when I leave here.”*  ***Participant 10:*** *“I recuperate pretty quick. But if I sit down and watch CNN or Fox or whatever, I’ll just fall asleep. For, for not a long time, maybe 10 minutes, that’s it.”* |
